# Age-Dependent Effects of Butyl Benzyl Phthalate Exposure on Lipid Metabolism and Hepatic Fibrosis in Mice

**DOI:** 10.3390/cells14020126

**Published:** 2025-01-16

**Authors:** Min-Seo Park, Seonhwa Hwang, Hyun-Bon Kang, Minjeong Ha, Juyeon Park, So-Youn Park, Yong-Joo Park, Min-Hi Park

**Affiliations:** 1 College of Pharmacy, Kyungsung University, 309 Suyeong-ro, Busan 48434, Republic of Korea; minseo829@naver.com (M.-S.P.); reseon17@naver.com (S.H.); rkdguasqhs23@naver.com (H.-B.K.); mini3882@naver.com (M.H.); a8230@naver.com (J.P.); 2 Brain Busan 21 Plus Research Project Group, Kyungsung University, Busan 48434, Republic of Korea; 3 Department of Pharmaceutical Science and Technology, Kyungsung University, Busan 48434, Republic of Korea; soyeonrang5811@gmail.com

**Keywords:** endocrine-disrupting chemicals (EDCs), butyl benzyl phthalate, non-alcoholic fatty liver disease, hepatic fibrosis, aging

## Abstract

Endocrine-disrupting chemicals (EDCs), including phthalates, have been implicated in the development of non-alcoholic fatty liver disease (NAFLD) and hepatic fibrosis. This study investigates the age-dependent effects of butyl benzyl phthalate (BBP) exposure on lipid metabolism in the livers of young and aged mice. Young (2-month-old) and aged (20-month-old) male C57BL/6 mice were exposed to BBP through drinking water at a dose of 169 μg/kg/day for 6 and 4 months, respectively. Young mice exposed to BBP showed fatty liver, with downregulation of key fatty acid oxidation genes (CPT1A, CPT1B, CPT2, and Acox1) and elevated pro-inflammatory cytokines (TNF-α and IL-6). In contrast, aged mice exhibited hepatic fibrosis, with increased collagen deposition and upregulation of genes related to fibrosis (Acta2, MMP2, TGF-ß1, and Col1a2), cirrhosis (CXCR4, SOX9, DCN, and MFAP4), and cancer (Bcl2, CDKN2a, c-Myc, and Fn1). Overall, these findings emphasize the importance of age when evaluating the risks of EDC exposure, such as BBP. Future research should focus on understanding the molecular mechanisms behind these age-related differences and explore Grem1 and SOCS3 as potential therapeutic targets for treating EDC-induced and age-related liver diseases.

## 1. Introduction

Endocrine-disrupting chemicals (EDCs) are exogenous compounds that disrupt hormonal signaling and regulation, leading to a wide range of adverse health effects in exposed organisms and their offspring [1,2,3]. These synthetic and naturally occurring chemicals are extensively used in various industrial and household products, resulting in near-ubiquitous human exposure throughout the human lifespan [4,5]. Common sources include medical devices, electronics, building materials, processed foods, and toys, reflecting their pervasive presence in contemporary environments [6].

Phthalates, a major class of EDCs, are extensively utilized as plasticizers to enhance the flexibility and durability of plastics [7]. Among them, butyl benzyl phthalate (BBP) is commonly used in products such as polyvinyl chloride (PVC), adhesives, and coatings [8]. Due to its non-covalent binding to materials, BBP readily migrates into the environment through evaporation, leaching, and surface degradation [9]. Consequently, BBP has been detected in environmental matrices such as air, water, and soil, with concentrations ranging from nanograms to micrograms per liter [10]. Human exposure to BBP occurs primarily through ingestion of contaminated food and water, inhalation of environmental pollutants, and dermal absorption, with daily intake levels estimated to range from 2 µg/kg/day in the general population to as high as 286 µg/kg/day in occupational settings [11]. Despite regulations in several countries, BBP and its metabolites persist in the environment, raising significant concerns about their potential long-term effects on human health [12,13]. Emerging evidence suggests that BBP exposure is associated with various toxic effects, including reproductive, developmental, and metabolic disruptions [14,15]. However, its specific role in liver diseases, particularly non-alcoholic fatty liver disease (NAFLD), remains poorly understood.

NAFLD, which affects approximately 25% of the global population, is characterized by the excessive accumulation of lipids in hepatocytes [16]. This condition progresses from steatosis to severe liver damage, such as fibrosis and cirrhosis, driven by mechanisms like oxidative stress and lipotoxicity [17,18]. While obesity and diabetes are well-established risk factors, increasing evidence emphasizes the role of environmental factors, such as EDCs, in exacerbating NAFLD by disrupting lipid metabolism and promoting hepatic dysfunction [19,20].

BBP’s potential contribution to NAFLD is particularly concerning, given its ability to impair lipid and glucose metabolism through mechanisms involving endocrine disruption and mitochondrial dysfunction [21,22]. Additionally, age-related differences in metabolic capacity, hormonal regulation, and detoxification processes may influence susceptibility to BBP-induced toxicity [23,24]. However, few studies have investigated how BBP exposure interacts with age to modulate NAFLD progression or exacerbate liver dysfunction. This study aims to address these gaps by examining the effects of BBP exposure on lipid accumulation and liver function in young and aged mice. By focusing on age-specific differences, this research intends to explore the mechanisms underlying BBP-induced NAFLD and its broader implications for liver health.

## 2. Materials and Methods

### 2.1. Materials

Benzyl butyl phthalate (Sigma-aldrich, Burlington, MA, USA); Dimethyl sulfoxide (Sigma-aldrich, Burlington, MA, USA); the EZ-Triglyceride Quantification Assay Kit (DoGenBio, Seoul, Republic of Korea); Formalin solution, neutral buffered, 10% (Sigma-aldrich, Burlington, MA, USA); Tissue-Tek^®^ O.C.T Compound (SAKURA, Tokyo, Japan); the Total RNA Extraction Kit (SMART GENE, Daejeon, Republic of Korea); the Compact cDNA Synthesis kit (SMART GENE, Daejeon, Republic of Korea); SYBR Green Master Mix (SMART GENE, Daejeon, Republic of Korea); and the RT² Profiler™ PCR Array mouse fibrosis (QIAGEN, Hilden, Germany) were obtained for this study.

### 2.2. Animals and BBP Treatment

Male C57BL/6 mice (7 weeks old) were obtained from the Korea Central Laboratory Animal Center (Seoul, Republic of Korea). A total of 24 mice were housed in an individually ventilated cage (IVC) system (Pure Palace; Raonbio, Yongin-si, Republic of Korea) under controlled environmental conditions: temperature (22 ± 1 °C), humidity (50 ± 5%), and a 12 h light/dark cycle. Standard chow and water were provided ad libitum. After a one-week acclimation period, the 24 mice were randomly divided into four groups (*n* = 6 per group): Young Control, Young BBP-Exposed, Old Control, and Old BBP-Exposed. Assuming a standard deviation of 5 for the measure and expecting a difference of at least 20 between the control and test groups, a non-parametric test required 6 animals in each group at a 5% significance level and 80% statistical power. The Young groups were sacrificed at 8 months, while the Old groups were sacrificed at 24 months of age. The timing of the sacrifice was based on the life cycle of the C57BL6 mouse. Mice at eight weeks old are considered to be in young adulthood, while those at 20 months old are regarded as elderly [25,26]. To evaluate the effects of prolonged BBP exposure at different life periods, BBP was administered to the Young BBP-Exposed group starting from 2 months of age for 6 months, while the Old BBP-Exposed group received BBP from 20 months of age for 4 months.

BBP exposure was administered by diluting BBP in drinking water, allowing for voluntary ingestion. The dosage of BBP was set at 169 μg/kg/day, calculated and adjusted monthly based on body weight and water intake measurements to ensure accurate exposure levels. The Young BBP-Exposed group received BBP from 2 months of age for 6 months, whereas the Old BBP-Exposed group received BBP from 20 months of age for 4 months. During the study, 5 mice from the treatment group were excluded from the analysis due to unexpected death. Liver tissues and blood from the mice were randomly numbered for the following experiments. Only the corresponding author was aware of the group allocation during the stages of conducting the experiment, the outcome assessment, and the data analysis. The experiments were conducted under the Institutional Animal Care and Use Committee (IACUC) Standard Operating Guidelines. The animal study protocol was approved by the Institutional Animal Care and Use Committee of Kyungsung University (Approval No. Re-search-21-020B; approval date: 26 January 2023).

### 2.3. Measurements of GOT and GPT Levels

Blood samples were collected, and serum was separated by centrifugation at 3000 rpm for 10 min and stored at −80 °C until analysis. Serum levels of aspartate aminotransferase (GOT) and alanine aminotransferase (GPT) were measured using commercial assay kits (Asan Pharm, Seoul, Republic of Korea) according to the manufacturer’s instructions. Absorbance was measured at 505 nm using a BioTek™ Epoch™ Microplate Spectrophotometer (Agilent Technologies, Santa Clara, CA, USA). Enzyme activities were calculated using standard curves derived from known concentrations.

### 2.4. Measurement of Triglyceride Levels

Hepatic triglyceride (TG) contents were measured using the EZ-Triglyceride Quantitative Assay Kit. Liver tissues (40–60 mg) were homogenized in 1 mL of 5% NP-40 solution (ThermoFisher Scientific, Waltham, MA, USA) on ice. The homogenates were heated to 80–100 °C for 5 min to solubilize triglycerides, cooled to room temperature, and reheated for an additional 5 min. After cooling, the samples were centrifuged at 13,000 rpm for 2 min to remove insoluble material, and the supernatants were collected. Assay was performed in 96-well plates by adding the provided buffer, reaction mixture, and samples according to the manufacturer’s protocol. Following a 30 min incubation in the dark, absorbance was measured at 570 nm using the BioTek™ Epoch™ Microplate Spectrophotometer. To normalize TG levels, the optical density (OD) readings were divided by the protein concentration of each sample, accounting for potential variations in tissue weight.

### 2.5. Histology Analysis (Oil Red O Staining and Picrosirius Red Staining)

To assess lipid accumulation and fibrosis in the liver, Oil red O staining and Picrosirius Red staining were performed, respectively. Liver tissues were fixed in 10% formalin overnight and washed with phosphate-buffered saline (PBS). Fixed tissues were embedded in Tissue-Tek^®^ O.C.T. Compound (SAKURA, Tokyo, Japan) and cryo-sectioned at a thickness of 4 μm. Sections were stained with oil red O to visualize lipid droplets and picrosirius red to visualize denatured collagen in tissues. The relative intensities of Oil Red O and Picrosirius Red staining were quantified using SlideViewer 2.5 software (3DHISTECH, Budapest, Hungary).

### 2.6. Quantitative Reverse Transcriptase Polymerase Chain Reaction (qRT-PCR)

Total RNA was extracted from the livers (10 mg) using the Total RNA Extraction Kit following the manufacturer’s instructions. RNA concentration and purity were assessed using a Nano-300 spectrophotometer. Complementary DNA (cDNA) was synthesized from 400 ng of total RNA using the Compact cDNA Synthesis Kit. Gene expression levels were quantified by qRT-PCR using the SYBR Green Kit on a QuantStudio 1 Real-Time PCR System (ThermoFisher Scientific). Primers specific for genes involved in fatty acid synthesis, fatty acid oxidation, cholesterol synthesis, and glucose metabolism were used (provided in Supplementary Table S1). The results were normalized to the average of 18S, Gusb, B2m, and GAPDH as an internal reference, and relative expression was calculated using the 2^−∆∆Ct^ method.

### 2.7. RT² Profiler™ PCR Array

PCR array was performed using the RT² Profiler PCR Array, which contains 84 specific genes related to mouse fibrosis. After quantifying the extracted RNA, cDNA was prepared using the RT² First Strand Kit. Then, PCR components, including RT² SYBR Green Mastermix, cDNA, and DNAse-/RNase-free water, were added to each well according to the manufacturer’s instructions. Reverse transcription reactions were carried out in Quant Studio 1 applied biosystems (ThermoFisher Scientific). The data were analyzed using a web-based tool provided by the GeneGlobe Data Analysis Center. The sequences of gene included in each well of the PCR array are shown in Supplementary Table S2.

### 2.8. Western Blot Analysis

Protein expression levels were determined with hepatic tissues. Cytoplasmic extracts were isolated using the EpiQuik^TM^ Nuclear Extraction Kit (EpigenTek, Farmingdale, NY, USA). The Pierce^TM^ BCA Protein Assay Kit (ThermoFisher Scientific) determined protein concentrations in the fractions. Samples were separated on 10% SDS-PAGE and electrophoretically transferred to PVDF membranes (Merck Millipore, Burlington, MA, USA) at 100 V. After blocking, the membranes were incubated with specific primary antibodies (1:1000 dilution; GREM1 Rabbit pAb, SOCS3 Rabbit mAb, Abclonal, Wuhan, China) overnight at 4 °C in a shaker. The next day, the membranes were incubated with IRDye^®^ 680RD Goat anti-Rabbit antibody (1:10,000 dilution) (LI-COR, Lincoln, NE, USA) for 1 h 30 min at RT in a shaker. Then, the protein bands were detected using the Odyssey^®^ XF Imaging System (LI-COR).

### 2.9. Statistical Analysis

Data are presented as means ± standard errors of the means (SEMs). Statistical significance between experimental groups was determined by one-way ANOVA with Tukey’s multiple comparison test and two-way ANOVA with Bonferroni using Prism 5 software (GraphPad Software, Inc., La Jolla, CA, USA), depending on the experiment. Differences were considered statistically significant at *p* < 0.05.

## 3. Results

### 3.1. Age-Dependent Effects of BBP on Body Weight, Water, and Food Intake

**Figure 1:**
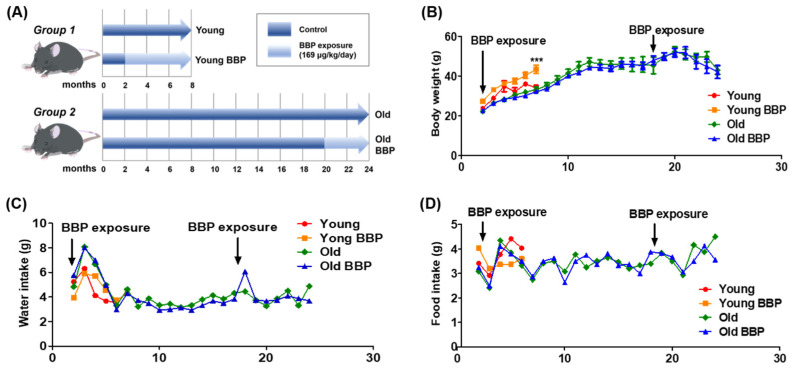
Establishment of mice exposed to BBP and natural aging animal model. C57BL/6 mice were divided into Young and Old groups. After that, Young BBP and Old BBP groups were formed: Young (*n* = 6), Young BBP (*n* = 5), Old (*n* = 4), and Old BBP (*n* = 4). (**A**) Two-month-old or twenty-month-old male mice ingested BBP with water for six months or four months. (**B**) Body weight changes of mice that ingested BBP with water and naive control mice (Young and Old groups) plotted over time. Intake of (**C**) water and (**D**) food in mice was measured monthly. Two-way ANOVA with Bonferroni was used to determine the significance of differences: *** *p* < 0.001 compared to the Young group.

The C57BL/6 mouse is a well-known laboratory model. These inbred mice are the primary mammalian model organisms due to their close genetic relationship to humans and their capability for genomic modification, allowing researchers to study human diseases. To investigate the effects of BBP on liver function in both young and old mice, we established a mouse model by exposing C57BL/6 mice to BBP through drinking water. Young mice (2 months old) were exposed to BBP for 6 months, while Old mice (20 months old) were exposed for 4 months (Figure 1A). Over the entire exposure period, the body weight of the BBP-treated mice exhibited changes compared to the Control groups. In young mice, BBP exposure resulted in a significant increase in body weight compared to the Young Control mice (*p* < 0.001) (Figure 1B). Despite these changes in body weight, no significant differences were observed in water or food intake between the BBP-Exposed and Control groups, indicating that the body weight differences were not due to variations in intake (Figure 1C,D). These findings suggest an age-dependent response to BBP exposure, with young mice showing a more pronounced susceptibility to increased body weight.

### 3.2. Effects of BBP on Liver Function and Toxicity

**Figure 2:**
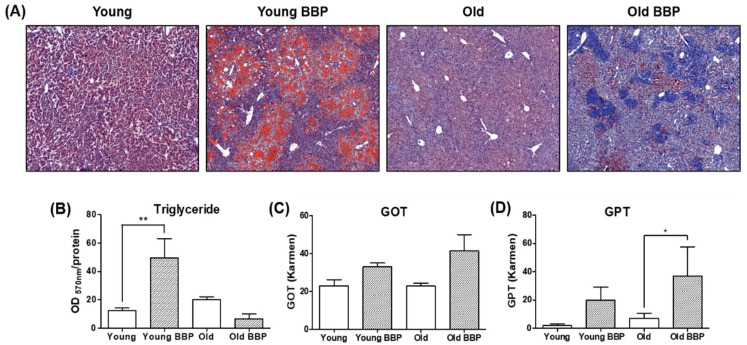
Liver enzyme concentration and liver steatosis status of the mice in each group. (**A**) Oil red O staining (10.0×). (**B**) Liver triglyceride (TG) contents of mice. (**C**) GOT and (**D**) GPT serum concentrations. One-way ANOVA with Bonferroni was used to determine the significance of differences: * *p* < 0.05 and ** *p* < 0.01 compared to Young or Old group.

Next, we examined the effect of BBP on liver enzyme levels and lipid accumulation. As shown in Figure 2A, the Oil Red O staining revealed a marked increase in lipid accumulation in the livers of the Young BBP-Exposed mice compared to the Young Control group. In contrast, the Old BBP-Exposed mice showed no significant difference compared to the Old Control group. Consistent with the Oil Red O staining results, liver triglyceride (TG) levels were significantly elevated in the Young BBP-Exposed group compared to the Young Control group (*p* < 0.01), indicating increased lipid accumulation in the liver (Figure 2B). Additionally, the levels of GOT and GPT in the BBP-Exposed groups were significantly elevated compared to the Control groups, with a more pronounced increase observed in the Old BBP-Exposed mice (Figure 2C,D). Interestingly, although Old BBP-Exposed mice showed elevated liver enzyme levels, there was no significant increase in liver TG contents compared to the Old Control group. These findings suggest that BBP exposure leads to lipid accumulation in the liver, particularly in young mice, while older mice experience more severe liver enzyme elevation without substantial lipid accumulation.

### 3.3. Effects of BBP on Lipid Metabolism and Pro-Inflammatory Cytokines in Young Mice

**Figure 3:**
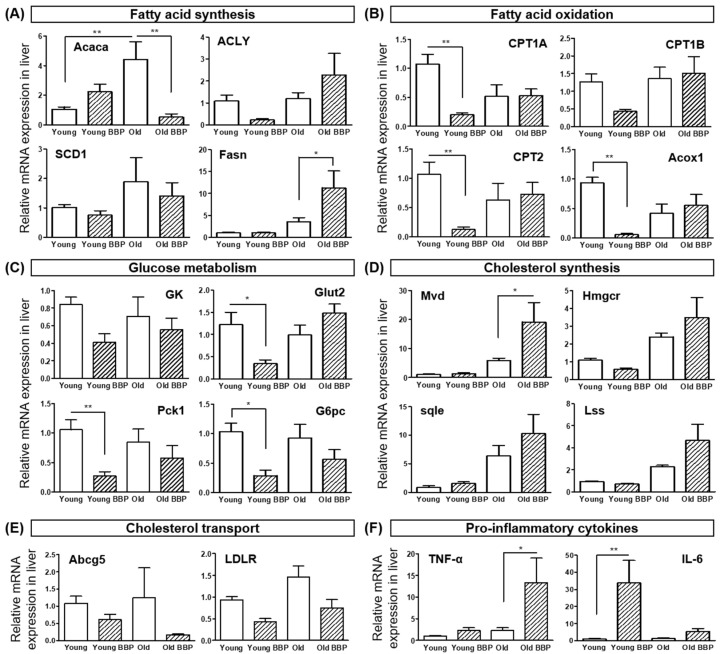
The effect of BBP exposure on the expression of metabolism-related genes in the liver tissues of Young and Old mice. (**A**) Expression of genes involved in fatty acid synthesis (Acaca: acetyl-CoA carboxylase alpha, Acly: ATP-citrate lyase, Scd1: stearoyl-CoA desaturase-1, and Fasn: fatty acid synthase). (**B**) Expression of genes involved in fatty acid oxidation (CPT1A: carnitine palmitoyltransferase 1a, CPT1B: carnitine palmitoyl transferase 1b, Cpt2: carnitine palmitoyltransferase 2, and Acox1: acyl-Coenzyme A oxidase 1). (**C**) Expression of genes involved in glucose metabolism (Gk: glucokinase, Glut2: glucose transporter 2, Pck1: phosphoenolpyruvate carboxykinase, and G6pc: glucose-6-phosphatase). (**D**) Expression of genes involved in cholesterol synthesis (Mvd: mevalonate decarboxylase, Hmgcer: Hmg- coenzyme A reductase, Sqle: squalene epoxidase, and Lss: lanosterol synthase). (**E**) Expression of genes involved in Cholesterol transport (Abcg5: ATP Binding Cassette Subfamily G Member 5 and LDLR: Low-Density Lipoprotein Receptor). (**F**) Expression of genes involved in pro-inflammatory cytokines (TNF-α: Tumor Necrosis Factor-alpha and IL-6: Interleukin 6). Tukey’s multiple comparison test with one-way ANOVA was used to confirm the significance of differences: * *p* < 0.05, and ** *p* < 0.01.

Furthermore, we examined the relative mRNA expression levels of key processes related to lipid metabolism, including fatty acid synthesis (Acaca, Acly, Scd1, and Fasn), fatty acid oxidation (Acox1, CPT1A, CPT1b, and Cpt2), glucose metabolism (Gk, G6pc, Glut2, and Pck1), cholesterol synthesis (Hmgcr, Lss, Mvd, and Sqle), and cholesterol transport (Abcg5 and LDLR). BBP exposure did not significantly affect the expression of genes related to fatty acid synthesis in the Young group. However, in the Old group, BBP exposure significantly increased the expression of Fasn, indicating an age-dependent effect of BBP on fatty acid synthesis (Figure 3A). Interestingly, however, BBP exposure significantly reduced the expression levels of key fatty acid oxidation genes, including CPT1A (*p* < 0.01), Cpt2 (*p* < 0.01), and Acox1 (*p* < 0.01), in young mice (Young BBP) compared with the Control group (Young), indicating impaired fatty acid oxidation (Figure 3B). In terms of glucose metabolism, BBP exposure led to a significant reduction in the expression of Gk (*p* < 0.05), Glut2 (*p* < 0.05), Pck1 (*p* < 0.01), and G6pc (*p* < 0.05) in Young mice (Young BBP) compared with the Control group (Young) (Figure 3C). For cholesterol synthesis-related genes (Hmgcr, Lss, Mvd, and Sqle) and cholesterol transport-related genes (Abcg5 and LDLR), no significant changes were observed in response to BBP exposure (Figure 3D,E). These results suggest that BBP exposure affects lipid metabolism primarily by inhibiting fatty acid oxidation in young mice, potentially contributing to lipid accumulation. Further, pro-inflammatory cytokines, such as TNF-α and IL-6, which contribute to metabolic dysregulation and promote lipid accumulation in the liver, were also examined to assess the inflammatory response induced by BBP exposure (Figure 3F). TNF-α (*p* < 0.05) and IL-6 (*p* < 0.01) expression were significantly elevated in the BBP-Exposed group compared to the Old and Young mouse groups. These results indicate an enhanced inflammatory response that may have contributed to the observed lipid accumulation in Young mice.

### 3.4. Effects of BBP on Hepatic Fibrosis in Aged Mice

**Figure 4:**
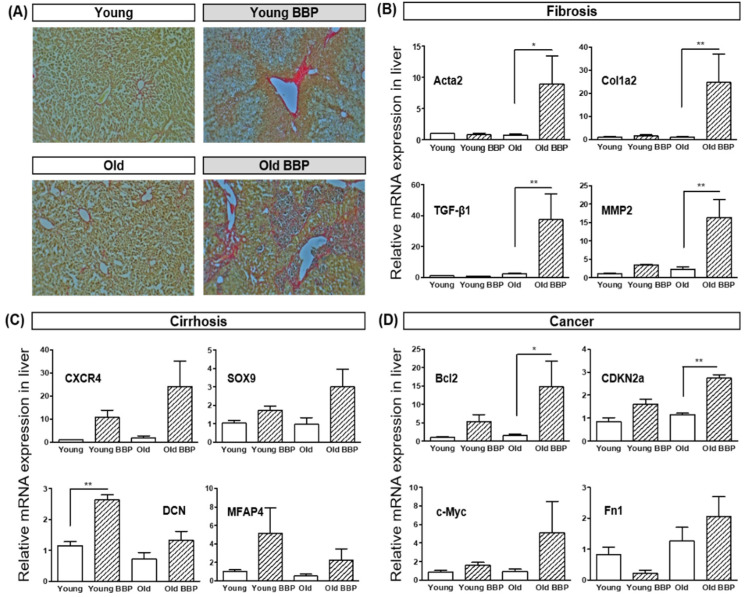
The effect of BBP exposure on the expression of NAFLD-related genes in liver tissue. (**A**) Liver fibrosis status of each group determined with Picrosirius Red (10.0×) staining. (**B**) Expression of genes involved in fibrosis (Acta2: actin alpha 2, MMP2: matrix metallopeptidase 2, TGF-β1: transforming growth factor beta 1, and Col1a2: collagen type I alpha 2 chain). (**C**) Expression of genes involved in cirrhosis (CXCR4: C-X-C motif chemokine receptor 4, SOX9: SRY-box transcription factor 9, DCN: decorin, and MFAP4: microfibril associated protein 4). (**D**) Expression of genes involved in cancer (Bcl2: B-cell leukemia/lymphoma-2, CDKN2a: cyclin-dependent kinase inhibitor 2a, c-Myc: MYC proto-oncogene, BHLH Transcription Factor, and Fn1: Fibronectin 1). Tukey’s Multiple comparison test with one-way ANOVA was used to confirm the significance of differences: * *p* < 0.05, and ** *p* < 0.01.

Hepatic fibrosis was observed to be a significant difference among the Old BBP-Exposure groups during dissection, and we performed Picrosirius Red staining (Figure 4A). The Old Control group displayed moderate collagen deposition, consistent with age-related fibrosis, while the Old BBP-Exposed group exhibited a marked increase in collagen deposition, suggesting advanced fibrosis due to BBP exposure.

Furthermore, we examined the relative mRNA expression levels of key markers related to hepatic fibrosis (Acta2, Cola2, TGF-β1, and MMP2), cirrhosis (CXCR4, SOX9, DCN, and MFAP4), and cancer (Bcl2, CDKN2a, c-Myc, and Fn1) across the experimental groups.

BBP exposure in the Old group tended to increase the expression of genes associated with hepatic fibrosis. Among them, the expression of Acta2 and MMP2 were significantly increased in the Old BBP group compared to the Old Control group (*p* < 0.01), indicating that BBP exposure leads to enhanced fibrotic progression in older animals. Although not significant, there was also a tendency for genes associated with cirrhosis and liver cancer to increase when BBP was administered to the Old group. BBP exposure may contribute to hepatic fibrosis, cirrhosis, and tumorigenic processes, particularly in old mice (Figure 4B–D).

### 3.5. Possible Molecular Mechanisms of BBP in the Fatty Liver

**Figure 5:**
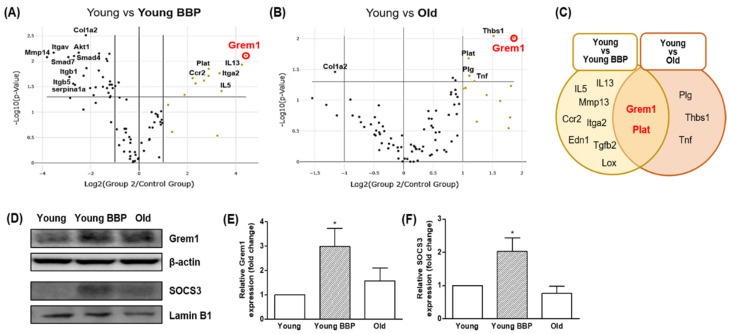
Comparison of gene expression in RT^2^ Profiler PCR Array Mouse Fibrosis. (**A**) Volcano plot comparing the Young group with the Young BBP group. (**B**) Volcano plot comparing the Young group with the Old group. (**C**) Venn diagram of overexpressed genes in Young vs. Young BBP and Young vs. Old. (**D**) Western blot analysis showing expression of Grem1 and SOCS3. Representative Western blot images of Grem1 and SOCS3 (*n* = 4). (**E**) Quantitative results for Grem1. (**F**) Quantitative results for SOCS3. Tukey’s multiple comparison test with one-way ANOVA was used to confirm the significance of differences: * *p* < 0.05 compared to Young group.

The RT² Profiler™ PCR Array was performed to identify gene expression related to hepatic fibrosis in our liver samples. We compared the expression levels of fibrosis-related genes in each group in the range of fold regulation above 2 and a *p*-value below 0.05. PCR array analysis compared the expression of 84 fibrosis-related genes in the Young vs. Young BBP group and the Young vs. Old group. (Precise values are shown in the Supplementary Tables S3 and S4.) In the Young vs. Young BBP comparison (left panel) (Figure 5A), several fibrosis-related genes, such as Grem1, IL13, IL5, and Ccr2, were upregulated in response to BBP exposure. Notably, Grem1 showed significant upregulation, suggesting its potential involvement in the lipid accumulation induced by BBP in young mice. Additionally, genes related to inflammation and extracellular matrix remodeling, such as MMP13, Itga2, and Tgfb2, were also upregulated, further indicating BBP’s role in promoting fibrosis.

In the Young vs. Old comparison (middle panel) (Figure 5B), age-related changes in gene expression showed upregulation of fibrosis-related genes, including Grem1, Thbs1, Tnf, and Plg, indicating that aging independently induces fibrotic processes. The upregulation of Grem1 in both the BBP-Exposed and Old groups suggests that this gene plays a central role in fibrosis, whether driven by environmental factors or during aging.

The Venn diagram summarizes the overlap between the two comparisons, showing that Grem1 and Plat were significantly upregulated in both the Young vs. Young BBP and Young vs. Old groups (Figure 5C). These findings suggest that Grem1 is a critical mediator of fibrotic processes in both BBP exposure and age-related fatty liver.

Given that Grem1 expression was significantly upregulated in both the Young BBP-Exposed and Old Control groups, we further assessed the protein expression of Grem1 (Figure 5D,E). Additionally, because of the observed increase in lipid accumulation in the Young BBP group, as well as in the Old group, we measured the expression of SOCS3, a key transcription factor involved in the regulation of lipid accumulation. As shown in Figure 5D,E, the protein expression levels of Grem1 were significantly elevated in the Young BBP-Exposed group compared to the Young Control group (*p* < 0.05). Furthermore, SOCS3 expression was significantly higher in the Young BBP group compared to the Control group (*p* < 0.05). This suggests that Grem1 and SOCS3 may have contributed to the increased lipid accumulation observed in the Young BBP-Exposed mice.

## 4. Discussion

EDCs have been increasingly implicated in lipid accumulation and the development of NAFLD [27,28,29]. Given that exposure to EDCs such as di(2-ethylhexyl) phthalate (DEHP) and mono(2-ethylhexyl) phthalate (MEHP) has been linked to an increased risk of metabolic syndrome and hepatic steatosis [30,31,32], we investigated the effects of BBP exposure on liver function and metabolism in both young and aged mice. This study examined critical physiological and molecular changes related to liver dysfunction, particularly the differences between young and aged mice in response to BBP exposure. Our findings revealed that young mice under BBP exposure accumulated lipid droplets in the liver, while aged mice exhibited hepatic fibrosis. These age-dependent responses suggest that younger mice are more susceptible to BBP-induced alterations in lipid metabolism, leading to fatty liver formation, while aged mice may exhibit a predisposition toward hepatic fibrosis.

The results of this study demonstrate the differential effects of BBP exposure on liver function and lipid metabolism across age groups. Notably, triglyceride levels were significantly elevated in the Young BBP group compared to the Young Control group, while such an increase was insignificant in the Old BBP group. This finding suggests that younger mice may be more susceptible to BBP-induced lipid accumulation, potentially due to age-related differences in hepatic metabolic activity or detoxification mechanisms. These findings indicate the critical role of age in modulating the effects of EDCs, underscoring the need to evaluate age-specific sensitivities. Previous studies have reported that metabolic responses to EDCs are age-dependent, which aligns with our observations [33].

Consistent with our findings, earlier studies have demonstrated that EDCs affect lipid metabolism in the liver, which is crucial for energy balance and storage. A damaged liver often leads to the release of liver enzymes such as GOT and GPT, which is a crucial indicator of liver dysfunction. Research in both human [34,35] and animal models [36] has shown that exposure to substances like bisphenol A (BPA) and phthalates, such as DEHP and diisononyl phthalate (DINP), is associated with increased liver enzyme levels. Our results confirmed that BBP exposure upregulated serum GOT and GPT levels in the Young and Old BBP-Exposure groups. This disruption likely occurs through both genomic and non-genomic pathways regulating lipid metabolism. The alteration of these pathways leads to an imbalance in lipid uptake and fatty acid oxidation, promoting fat accumulation in liver tissue [37,38].

An imbalance between lipid uptake and oxidation primarily drives the accumulation of lipids in the liver [39]. In our study, young mice exposed to BBP showed enhanced lipid accumulation, supporting the hypothesis that BBP impairs fatty acid oxidation pathways while promoting fatty acid and cholesterol synthesis. Elevated triglyceride levels observed in histological and biochemical assessments further evidenced these metabolic disruptions. Our findings also revealed a suppression of fatty acid synthesis in the Old BBP group compared to the Young BBP group, which may reflect an age-dependent downregulation of lipid biosynthesis pathways. This suppression likely results in a reduced ability to store energy as lipids in older mice, contrasting with the enhanced lipid accumulation observed in younger mice. On the other hand, fatty acid oxidation, a key metabolic pathway for breaking down lipids, was significantly reduced in the Young BBP group. This reduction in β-oxidation, as evidenced by the downregulation of CPT1A and PGC1α, is likely a major contributor to lipid droplet accumulation in young mice. In contrast, aged mice maintained fatty acid oxidation, suggesting a compensatory mechanism that may protect against lipid accumulation but predispose them to oxidative stress and fibrosis. Similar lipid accumulation patterns have also been documented in research on mice exposed to EDCs [40]. Moreover, histological analysis of aged mice exposed to BBP revealed an induction of hepatic fibrosis, a hallmark of advanced liver injury instead of lipid accumulation. The fibrosis observed in the aged BBP group underscores the age-dependent effects of BBP exposure, where young mice tend to develop fatty liver. In contrast, aged mice are more likely to progress toward a fibrotic phenotype.

Our results indicate that the downregulation of genes related to gluconeogenesis and β-oxidation is closely associated with developing fatty liver in young mice exposed to BBP. This disruption of key metabolic pathways likely impairs the liver’s ability to maintain energy homeostasis, resulting in lipid accumulation. Additionally, the suppression of fatty acid synthesis genes, coupled with reduced fatty acid oxidation, further demonstrated the multifaceted disruption of lipid metabolism in the Young BBP group. This combination of impaired biosynthesis and oxidation pathways may exacerbate lipid storage and promote the observed metabolic imbalance. These findings are consistent with previous studies reporting reduced expression of mitochondrial β-oxidation-related genes, such as PPARα, PGC1α, and CPT1A, in patients with fatty liver [24,41,42,43]. Furthermore, the role of gluconeogenesis in fatty liver development has been well documented, with the downregulation of critical genes, including PEPCK and G6Pase, having been shown to decrease glucose production while promoting lipid accumulation in the liver [44,45]. These results emphasize the crucial role of metabolic gene regulation in maintaining liver health and suggest that targeting the pathways involved in gluconeogenesis and β-oxidation may provide therapeutic strategies for preventing BBP-induced fatty liver.

Similarly, studies on DEHP have shown a comparable downregulation of fatty acid oxidation genes, resulting in lipid accumulation in the liver and contributing to NAFLD. DEHP is also known to inhibit the expression of the CPT1 gene, further impairing fatty acid oxidation. In addition to impaired fatty acid oxidation, our findings showed that TNF-α and IL-6, cytokines known to play important roles in promoting lipid accumulation, were significantly elevated in the BBP-Exposed Young mice. TNF-α [46] and IL-6 promote inflammation and lipogenesis [47,48]. These elevated cytokines suggest that both inflammation and disrupted oxidation contribute to the observed lipid accumulation in young mice.

Elevated Grem1 levels have been linked to increased inflammation and fibrosis in the liver, contributing to the progression of NAFLD. Previous studies have established Grem1 as a critical regulator in the development of NAFLD and liver fibrosis by its inhibition of bone morphogenetic protein (BMP) signaling [49,50]. Our study observed a significant upregulation of Grem1 in young BBP-exposed mice, indicating that BBP may accelerate NAFLD development. Furthermore, Grem1 expression was elevated in both young BBP-exposed and aged mice, highlighting its dual role in BBP-induced liver damage and age-related lipid accumulation. These findings emphasize the importance of Grem1 as a mediator in NAFLD progression and underscore its potential as a therapeutic target for addressing both toxin-induced and age-related NAFLD.

SOCS3 (Suppressor of Cytokine Signaling 3) is primarily known for regulating insulin signaling and inflammation, which can indirectly impact lipid oxidation and glucose metabolism [51,52,53]. By inhibiting insulin signaling, SOCS3 can promote lipid accumulation and contribute to NAFLD development [54]. Previous studies have shown that overexpression of SOCS3 is associated with impaired fatty acid oxidation and glucose metabolism, contributing to the progression of metabolic disorders [55,56]. In this study, young BBP-exposed mice exhibited disrupted pathways regulated by SOCS3, which led to impaired fatty acid oxidation and increased lipid accumulation. However, aged mice exposed to BBP did not show significant changes in fatty acid oxidation genes or SOCS3 expression, suggesting a potential age-dependent variation in BBP’s effects on lipid metabolism. Despite these findings, the precise interrelationships between SOCS3 and genes involved in fatty acid oxidation and lipid accumulation remain unclear. Further experiments are necessary to explore these mechanisms in detail. Additionally, the limited availability of serum samples prevented glucose metabolism measurements, such as glucose or insulin levels, from validating the observed gene expression changes. This limitation determines the need for future studies incorporating both functional assays and metabolic analyses to provide a more comprehensive understanding of the mechanisms underlying SOCS3-related lipid accumulation and BBP-induced fatty liver.

## 5. Conclusions

In conclusion, this study highlights the age-dependent effects of BBP exposure, with young mice demonstrating a predisposition to fatty liver and aged mice showing evidence of hepatic fibrosis. These findings underscore the need to consider both age and exposure duration when assessing the hepatotoxicity of EDCs. Future studies should focus on identifying specific molecular targets, such as Grem1 and SOCS3, that drive BBP-induced lipid accumulation in young livers to develop therapeutic strategies for toxin-induced and age-related liver diseases. Additionally, further investigations are also needed to explore the molecular mechanisms underlying fibrosis in aged BBP-exposed mice. Although C57BL/6 mice have been useful in previous studies of liver fibrosis, there may still be differences in response to exogenous agents between species. Therefore, further studies using clinical analyses or human-derived cell lines would help understand the results and mechanisms obtained in this study.

## Data Availability

The data presented in this study are available on request from the corresponding author.

## Supplementary Materials

The following supporting information can be downloaded at www.mdpi.com/article/10.3390/cells14020126/s1: Table S1: Primer sequences used in qRT-PCR analysis; Table S2: Gene list of RT^2^ PCR array; Table S3: RT^2^ Profiler PCR array fold change (Young vs. Young BBP); Table S4: RT^2^ Profiler PCR array fold change (Young vs. Old).
