# Supplementary material for: Age-Dependent Effects of Butyl Benzyl Phthalate Exposure on Lipid Metabolism and Hepatic Fibrosis in Mice
Source: Cells. 2025 Jan 16;14(2):126. doi: 10.3390/cells14020126 (PMC11764096; doi:10.3390/cells14020126)
Supplement: Supplementary file 1 [file cells-14-00126-s001.zip › cells-3415737-supplementary.pdf]

Supplemental Table S1. Primer sequences used in qRT-PCR analysis.

**Fatty acid synthesis**	m-Acaca F	CTCCCGATTCATAATTGGGTCTG	**Cholesterol transport**	m-Abcg5 F	AGGGCCTCACATCAACAGAG
	m-Acaca R	TCGACCTTGTTTTACTAGGTGC		m-Abcg5 R	GCTGACGCTGTAGGACACAT
	m-Acly F	CAGCCAAGGCAATTTCAGAGC		m-LDLR F	TGACTCAGACGAACAAGGCTG
	m-Acly R	CTCGACGTTTGATTAACTGGTCT		m-LDLR R	ATCTAGGCAATCTCGGTCTCC
	m-SCD1 F	TTCTTGCGATACACTCTGGTGC	**Inflammatory cytokines**	m-TNFa F	CAGGCGGTGCCTATGTCTC
	m-SCD1 R	CGGGATTGAATGTTCTTGTCGT		m-TNFa R	CGATCACCCCGAAGTTCAGTAG
	m-Fasn F	GGAGGTGGTGATAGCCGGTAT		m-IL6 F	GCAGCATCACCTTCGCTTAGA
	m-Fasn R	TGGGTAATCCATAGAGCCCAG		m-IL6 R	CAGATATTGGCATGGGAGCAAG
**Fatty acid oxidation**	m-Cpt1a F	CTCCGCCTGAGCCATGAAG	**Fibrosis**	m-Acta2(α-SMA) F	GTCCCAGACATCAGGGAGTAA
	m-Cpt1a R	CACCAGTGATGATGCCATTCT		m-Acta2(α-SMA) R	TCGGATACTTCAGCGTCAGGA
	m-Cpt1b F	GCACACCAGGCAGTAGCTTT		m-Mmp2 F	CAAGTTCCCCGGCGATGTC
	m-Cpt1b R	CAGGAGTTGATTCCAGACAGGTA		m-Mmp2 R	TTCTGGTCAAGGTCACCTGTC
	m-Cpt2 F	CCTGCTCGCTCAGGATAAACA		m-TGFβ1 F	CTCCCGTGGCTTCTAGTGC
	m-Cpt2 R	GTGTCTTCAGAAACCGCACTG		m-TGFβ1 R	GCCTTAGTTTGGACAGGATCTG
	m-Acox1 F	TAACTTCCTCACTCGAAGCCA		m-Col1a2 F	GTAACTTCGTGCCTAGCAACA
	m-Acox1 R	AGTTCCATGACCCATCTCTGTC		m-Col1a2 R	CCTTTGTCAGAATACTGAGCAGC
**Glucose metabolism**	m-Gk F	TGGGTAGAACAAGACCCGAAG	**Cirrhosis**	m-CXCR4 F	CTTCTGGGCAGTTGATGCCAT
	m-Gk R	GTTGCTGACACCAATGGCTT		m-CXCR4 R	CTGTTGGTGGCGTGGACAAT
	m-Glut2 F	TCAGAAGACAAGATCACCGGA		m-SOX9 F	AGTACCCGCATCTGCACAAC
	m-Glut2 F	GCTGGTGTGACTGTAAGTGGG		m-SOX9 F	ACGAAGGGTCTCTTCTCGCT
	m-Pck1 F	CTGCATAACGGTCTGGACTTC		m-DCN F	GACCCACAGGACGAAAACAAA
	m-Pck1 R	CAGCAACTGCCCGTACTCC		m-DCN R	GACCAAAACGCTGATACTGGC
	m-G6pc F	CGACTCGCTATCTCCAAGTGA		m-MFAP4 F	GCAACCCCTGGACTGTGATG
	m-G6pc R	GTTGAACCAGTCTCCGACCA		m-MFAP4 R	TTGTCATGTCGCAGAAGACGG
**Cholesterol synthesis**	m-Mvd F	CCGGTCAACATCGCAGTTATC	**Cancer**	m-Bcl-2 F	ATGCCTTTGTGGAACTATATGGC
	m-Mvd R	TTGTGGTCGTTTTTAGCTGGT		m-Bcl-2 R	GGTATGCACCCAGAGTGATGC
	m-Hmgcr F	AGAGCGAGTGCATTAGCAAAG		m-CDKN2A F	GCTCAACTACGGTGCAGATTC
	m-Hmgcr R	GATTGCCATTCCACGAGCTAT		m-CDKN2A R	GCACGATGTCTTGATGTCCC
	m-Sqle F	ATAAGAAATGCGGGGATGTCAC		m-c-Myc F	ATGCCCCTCAACGTGAACTTC
	m-Sqle R	ATATCCGAGAAGGCAGCGAAC		m-c-Myc R	CGCAACATAGGATGGAGAGCA
	m-Lss F	GGACTTACCAAAAGCGCAAAC		m-Fn1 F	ATGTGGACCCCTCCTGATAGT
	m-Lss R	GAAGAGCGGACCACCATAATC		m-Fn1 R	GCCCAGTGATTTCAGCAAAGG

Supplemental Table S2. Gene list of RT[2] PCR array.

Position	Unigene	Refseq	Symbol	Description
A01	Mm.213025	NM_007392	Acta2	Actin, alpha 2, smooth muscle, aorta
A02	Mm.301626	NM_007428	Agt	Angiotensinogen (serpin peptidase inhibitor, clade A, member 8)
A03	Mm.6645	NM_009652	Akt1	Thymoma viral proto-oncogene 1
A04	Mm.257460	NM_009741	Bcl2	B-cell leukemia/lymphoma 2
A05	Mm.595	NM_007557	Bmp7	Bone morphogenetic protein 7
A06	Mm.28278	NM_007616	Cav1	Caveolin 1, caveolae protein
A07	Mm.4686	NM_011330	Ccl11	Chemokine (C-C motif) ligand 11
A08	Mm.867	NM_011331	Ccl12	Chemokine (C-C motif) ligand 12
A09	Mm.1282	NM_011337	Ccl3	Chemokine (C-C motif) ligand 3
A10	Mm.6272	NM_009915	Ccr2	Chemokine (C-C motif) receptor 2
A11	Mm.439656	NM_009883	Cebpb	CCAAT/enhancer binding protein (C/EBP), beta
A12	Mm.277792	NM_007743	Col1a2	Collagen, type I, alpha 2
B01	Mm.249555	NM_009930	Col3a1	Collagen, type III, alpha 1
B02	Mm.390287	NM_010217	Ccn2	Cellular communication network factor 2
B03	Mm.1401	NM_009911	Cxcr4	Chemokine (C-X-C motif) receptor 4
B04	Mm.56769	NM_007833	Dcn	Decorin
B05	Mm.14543	NM_010104	Edn1	Endothelin 1
B06	Mm.252481	NM_010113	Egf	Epidermal growth factor
B07	Mm.225297	NM_007932	Eng	Endoglin
B08	Mm.3355	NM_010177	Fasl	Fas ligand (TNF superfamily, member 6)
B09	Mm.166318	NM_011824	Grem1	Gremlin 1
B10	Mm.267078	NM_010427	Hgf	Hepatocyte growth factor
B11	Mm.240327	NM_008337	Ifng	Interferon gamma
B12	Mm.874	NM_010548	Il10	Interleukin 10
C01	Mm.1284	NM_008355	Il13	Interleukin 13
C02	Mm.368330	NM_008356	Il13ra2	Interleukin 13 receptor, alpha 2
C03	Mm.15534	NM_010554	Il1a	Interleukin 1 alpha
C04	Mm.222830	NM_008361	Il1b	Interleukin 1 beta
C05	Mm.276360	NM_021283	Il4	Interleukin 4
C06	Mm.4461	NM_010558	Il5	Interleukin 5
C07	Mm.274846	NM_010562	Ilk	Integrin linked kinase
C08	Mm.3510	NM_008382	Inhbe	Inhibin beta E
C09	Mm.317280	NM_00103322 8	Itga1	Integrin alpha 1
C10	Mm.5007	NM_008396	Itga2	Integrin alpha 2
C11	Mm.57035	NM_013565	Itga3	Integrin alpha 3
C12	Mm.227	NM_008402	Itgav	Integrin alpha V
D01	Mm.263396	NM_010578	Itgb1	Integrin beta 1 (fibronectin receptor beta)
D02	Mm.87150	NM_016780	Itgb3	Integrin beta 3
D03	Mm.6424	NM_010580	Itgb5	Integrin beta 5
D04	Mm.98193	NM_021359	Itgb6	Integrin beta 6
D05	Mm.217000	NM_177290	Itgb8	Integrin beta 8
D06	Mm.275071	NM_010591	Jun	Jun oncogene

Position	Unigene	Refseq	Symbol	Description
D07	Mm.172	NM_010728	Lox	Lysyl oxidase
D08	Mm.269747	NM_019919	Ltbp1	Latent transforming growth factor beta binding protein 1
D09	Mm.5022	NM_008607	Mmp13	Matrix metallopeptidase 13
D10	Mm.280175	NM_008608	Mmp14	Matrix metallopeptidase 14 (membrane-inserted)
D11	Mm.156952	NM_032006	Mmp1a	Matrix metallopeptidase 1a (interstitial collagenase)
D12	Mm.29564	NM_008610	Mmp2	Matrix metallopeptidase 2
E01	Mm.4993	NM_010809	Mmp3	Matrix metallopeptidase 3
E02	Mm.16415	NM_008611	Mmp8	Matrix metallopeptidase 8
E03	Mm.4406	NM_013599	Mmp9	Matrix metallopeptidase 9
E04	Mm.2444	NM_010849	Myc	Myelocytomatosis oncogene
E05	Mm.256765	NM_008689	Nfkb1	Nuclear factor of kappa light polypeptide gene enhancer in B-cells 1, p105
E06	Mm.2675	NM_008808	Pdgfa	Platelet derived growth factor, alpha
E07	Mm.144089	NM_011057	Pdgfb	Platelet derived growth factor, B polypeptide
E08	Mm.154660	NM_008872	Plat	Plasminogen activator, tissue
E09	Mm.4183	NM_008873	Plau	Plasminogen activator, urokinase
E10	Mm.971	NM_008877	Plg	Plasminogen
E11	Mm.439692	NM_009243	Serpina1a	Serine (or cysteine) peptidase inhibitor, clade A, member 1a
E12	Mm.250422	NM_008871	Serpine1	Serine (or cysteine) peptidase inhibitor, clade E, member 1
F01	Mm.22708	NM_009825	Serpinh1	Serine (or cysteine) peptidase inhibitor, clade H, member 1
F02	Mm.152699	NM_010754	Smad2	MAD homolog 2 (Drosophila)
F03	Mm.7320	NM_016769	Smad3	MAD homolog 3 (Drosophila)
F04	Mm.100399	NM_008540	Smad4	MAD homolog 4 (Drosophila)
F05	Mm.325757	NM_008542	Smad6	MAD homolog 6 (Drosophila)
F06	Mm.34407	NM_001042660	Smad7	MAD homolog 7 (Drosophila)
F07	Mm.2093	NM_011427	Snai1	Snail homolog 1 (Drosophila)
F08	Mm.4618	NM_013672	Sp1	Trans-acting transcription factor 1
F09	Mm.277406	NM_009283	Stat1	Signal transducer and activator of transcription 1
F10	Mm.121721	NM_009284	Stat6	Signal transducer and activator of transcription 6
F11	Mm.248380	NM_011577	Tgfb1	Transforming growth factor, beta 1
F12	Mm.18213	NM_009367	Tgfb2	Transforming growth factor, beta 2
G01	Mm.3992	NM_009368	Tgfb3	Transforming growth factor, beta 3
G02	Mm.197552	NM_009370	Tgfbr1	Transforming growth factor, beta receptor I
G03	Mm.172346	NM_009371	Tgfbr2	Transforming growth factor, beta receptor II
G04	Mm.101034	NM_009372	Tgif1	TGFB-induced factor homeobox 1
G05	Mm.4159	NM_011580	Thbs1	Thrombospondin 1
G06	Mm.26688	NM_011581	Thbs2	Thrombospondin 2
G07	Mm.8245	NM_011593	Timp1	Tissue inhibitor of metalloproteinase 1
G08	Mm.206505	NM_011594	Timp2	Tissue inhibitor of metalloproteinase 2
G09	Mm.4871	NM_011595	Timp3	Tissue inhibitor of metalloproteinase 3
G10	Mm.255607	NM_080639	Timp4	Tissue inhibitor of metalloproteinase 4
G11	Mm.1293	NM_013693	Tnf	Tumor necrosis factor
G12	Mm.282184	NM_009505	Vegfa	Vascular endothelial growth factor A

Supplemental Table S3. RT^2 Profiler PCR array fold change (Young vs Young BBP).

Fold Change (comparing to Young group)							
Position	Symbol	Young BBP		Position	Symbol	Young BBP	
		Fold Change	p-value			Fold Change	p-value
A01	Acta2	0.20	0.088938	D07	Lox	6.43	0.023920
A02	Agt	0.25	0.033726	D08	Ltbp1	2.30	0.072135
A03	Akt1	0.18	0.006740	D09	Mmp13	3.69	0.045680
A04	Bcl2	0.57	0.051545	D10	Mmp14	0.07	0.008273
A05	Bmp7	0.78	0.738150	D11	Mmp1a	2.58	0.245571
A06	Cav1	0.45	0.157828	D12	Mmp2	1.89	0.096248
A07	Ccl11	9.44	0.291518	E01	Mmp3	1.95	0.163555
A08	Ccl12	1.15	0.579291	E02	Mmp8	1.71	0.123133
A09	Ccl3	0.97	0.833093	E03	Mmp9	0.77	0.831528
A10	Ccr2	7.33	0.019302	E04	Myc	1.35	0.200992
A11	Cebpb	1.55	0.159133	E05	Nfkb1	0.68	0.251764
A12	Col1a2	0.22	0.003057	E06	Pdgfa	0.35	0.020530
B01	Col3a1	0.65	0.598766	E07	Pdgfb	1.61	0.107566
B02	Ccn2	0.76	0.309888	E08	Plat	7.35	0.014042
B03	Cxcr4	1.35	0.336879	E09	Plau	0.56	0.447432
B04	Dcn	0.99	0.810602	E10	Plg	0.66	0.212510
B05	Edn1	5.05	0.027233	E11	Serpina1a	0.16	0.029059
B06	Egf	1.07	0.404471	E12	Serpine1	0.84	0.926458
B07	Eng	0.46	0.028797	F01	Serpinh1	0.43	0.051042
B08	Fasl	1.91	0.141330	F02	Smad2	0.42	0.041749
B09	Grem1	21.36	0.007765	F03	Smad3	0.78	0.344035
B10	Hgf	0.29	0.032384	F04	Smad4	0.13	0.008253
B11	Ifng	0.65	0.355512	F05	Smad6	0.32	0.006985
B12	Il10	0.84	0.964152	F06	Smad7	0.16	0.007853
C01	Il13	19.09	0.011629	F07	Snai1	0.35	0.031745
C02	Il13ra2	1.78	0.776642	F08	Sp1	0.43	0.028836
C03	Il1a	0.89	0.614580	F09	Stat1	0.47	0.033751
C04	Il1b	0.14	0.052636	F10	Stat6	0.40	0.009856
C05	Il4	1.70	0.113285	F11	Tgfb1	0.29	0.065877
C06	Il5	10.65	0.038475	F12	Tgfb2	4.65	0.021455
C07	Ilk	0.43	0.007082	G01	Tgfb3	0.20	0.019194
C08	Inhbe	0.44	0.048754	G02	Tgfbr1	0.40	0.033719
C09	Itga1	0.51	0.030699	G03	Tgfbr2	0.22	0.013611
C10	Itga2	10.06	0.017137	G04	Tgif1	0.78	0.403143
C11	Itga3	0.69	0.213092	G05	Thbs1	1.84	0.156434
C12	Itgav	0.09	0.006749	G06	Thbs2	1.47	0.201730
D01	Itgb1	0.14	0.020275	G07	Timp1	0.53	0.218029
D02	Itgb3	0.39	0.008987	G08	Timp2	0.43	0.024818
D03	Itgb5	0.15	0.027553	G09	Timp3	0.31	0.015122
D04	Itgb6	0.90	0.691825	G10	Timp4	1.87	0.175817
D05	Itgb8	0.27	0.046424	G11	Tnf	0.89	0.920650
D06	Jun	0.77	0.602236	G12	Vegfa	0.56	0.026043

Supplemental Table S4. RT^2 Profiler PCR array fold change (Young vs Old).

Fold Change (comparing to Young group)							
Position	Symbol	Old		Position	Symbol	Old	
		Fold Change	p-value			Fold Change	p-value
A01	Acta2	1.65	0.690301	D07	Lox	1.48	0.651646
A02	Agt	0.63	0.271870	D08	Ltbp1	1.41	0.277992
A03	Akt1	0.73	0.311202	D09	Mmp13	3.10	0.102640
A04	Bcl2	0.80	0.317738	D10	Mmp14	0.46	0.176907
A05	Bmp7	0.72	0.373213	D11	Mmp1a	3.48	0.190059
A06	Cav1	1.39	0.766354	D12	Mmp2	1.88	0.088622
A07	Ccl11	1.45	0.443729	E01	Mmp3	2.36	0.223507
A08	Ccl12	1.70	0.173178	E02	Mmp8	1.78	0.043405
A09	Ccl3	1.81	0.117964	E03	Mmp9	1.83	0.047536
A10	Ccr2	1.75	0.467834	E04	Myc	1.83	0.067846
A11	Cebpb	2.01	0.064800	E05	Nfkb1	1.13	0.554011
A12	Col1a2	0.45	0.034647	E06	Pdgfa	1.10	0.683724
B01	Col3a1	0.77	0.499679	E07	Pdgfb	1.74	0.080785
B02	Ccn2	1.39	0.259619	E08	Plat	2.14	0.020875
B03	Cxcr4	1.88	0.375982	E09	Plau	1.75	0.074349
B04	Dcn	1.08	0.996680	E10	Plg	2.16	0.040124
B05	Edn1	1.24	0.673007	E11	Serpina1a	1.86	0.167746
B06	Egf	1.09	0.526471	E12	Serpine1	1.36	0.791858
B07	Eng	1.46	0.147622	F01	Serpinh1	1.92	0.114705
B08	Fasl	2.64	0.082344	F02	Smad2	1.26	0.567502
B09	Grem1	3.63	0.009708	F03	Smad3	1.20	0.625521
B10	Hgf	0.60	0.186577	F04	Smad4	0.76	0.356315
B11	Ifng	0.93	0.584706	F05	Smad6	0.71	0.188491
B12	Il10	0.84	0.604834	F06	Smad7	0.55	0.177342
C01	Il13	1.27	0.297545	F07	Snai1	0.75	0.332619
C02	Il13ra2	3.42	0.285021	F08	Sp1	0.75	0.232083
C03	Il1a	1.46	0.334281	F09	Stat1	0.75	0.645450
C04	Il1b	0.98	0.373344	F10	Stat6	0.78	0.266291
C05	Il4	1.85	0.079936	F11	Tgfb1	0.61	0.227517
C06	Il5	1.34	0.198651	F12	Tgfb2	1.39	0.200434
C07	Ilk	0.84	0.509954	G01	Tgfb3	1.52	0.329056
C08	Inhbe	0.54	0.097842	G02	Tgfbr1	0.97	0.579920
C09	Itga1	1.04	0.902100	G03	Tgfbr2	1.12	0.999089
C10	Itga2	3.52	0.058650	G04	Tgif1	0.89	0.536836
C11	Itga3	0.99	0.988763	G05	Thbs1	2.86	0.009038
C12	Itgav	0.40	0.118958	G06	Thbs2	0.86	0.417008
D01	Itgb1	0.34	0.092879	G07	Timp1	1.61	0.644598
D02	Itgb3	1.16	0.623606	G08	Timp2	1.16	0.620166
D03	Itgb5	0.71	0.362172	G09	Timp3	0.68	0.246894
D04	Itgb6	1.14	0.580690	G10	Timp4	0.94	0.885556
D05	Itgb8	1.18	0.957848	G11	Tnf	2.29	0.048780
D06	Jun	1.01	0.891029	G12	Vegfa	0.94	0.667731
